# Insights Into *de novo* Mutation Variation in Lithuanian Exome

**DOI:** 10.3389/fgene.2018.00315

**Published:** 2018-08-14

**Authors:** Laura Pranckėnienė, Audronė Jakaitienė, Laima Ambrozaitytė, Ingrida Kavaliauskienė, Vaidutis Kučinskas

**Affiliations:** Department of Human and Medical Genetics, Institute of Biomedical Sciences, Faculty of Medicine, Vilnius University, Vilnius, Lithuania

**Keywords:** *de novo* mutation, mutation rate, Lithuanian, exome sequencing, population genetics

## Abstract

In the last decade, one of the biggest challenges in genomics research has been to distinguish definitive pathogenic variants from all likely pathogenic variants identified by next-generation sequencing. This task is particularly complex because of our lack of knowledge regarding overall genome variation and pathogenicity of the variants. Therefore, obtaining sufficient information about genome variants in the general population is necessary as such data could be used for the interpretation of *de novo* mutations (DNMs) in the context of patient’s phenotype in cases of sporadic genetic disease. In this study, data from whole-exome sequencing of the general population in Lithuania were directly examined. In total, 84 (VarScan) and 95 (VarSeq^TM^) DNMs were identified and validated using different algorithms. Thirty-nine of these mutations were considered likely to be pathogenic based on gene function, evolutionary conservation, and mutation impact. The mutation rate estimated per position pair per generation was 2.74 × 10^-8^ [95% CI: 2.24 × 10^-8^–3.35 × 10^-8^] (VarScan) and 2.4 × 10^-8^ [95% CI: 1.96 × 10^-8^–2.99 × 10^-8^] (VarSeq^TM^), with 1.77 × 10^-8^ [95% CI: 6.03 × 10^-9^–5.2 × 10^-8^] *de novo* indels per position per generation. The rate of germline DNMs in the Lithuanian population and the effects of the genomic and epigenetic context on DNM formation were calculated for the first time in this study, providing a basis for further analysis of DNMs in individuals with genetic diseases. Considering these findings, additional studies in patient groups with genetic diseases with unclear etiology may facilitate our ability to distinguish certain pathogenic or adaptive DNMs from tolerated background DNMs and to reliably identify disease-causing DNMs by their properties through direct observation.

## Introduction

Germline *de novo* mutations (DNMs) are genetic changes in the individual caused by mutagenesis occurring in parental gametes during oogenesis and spermatogenesis. Here, the term “*de novo*" should not be confused with the term “novel mutation." Despite the fact that DNMs in the context of a trio (father, mother, and child) are novel mutations, they may be common, rare, or novel variants in the general population. To measure and explain the rate of a particular DNM, it is necessary to assess the impact on phenotype of the variant first, because new favorable traits may evolve when arising genetic mutations offer a specific survival benefit (Front Line Genomics, 2017).

In humans with genetic non-Mendelian diseases that occur sporadically, DNMs are usually novel, more reliable, and more harmful than inherited variants because they are not subjected to strong natural selection (Crow, 2000; Front Line Genomics, 2017). Therefore, identifying the genetic cause of a disorder induced by a DNM in an individual can be challenging from a clinical standpoint, because pleiotropy and genetic heterogeneity may underlie a single phenotype (Eyre-Walker and Keightley, 2007). Accordingly, in the last decade, considerable efforts have been made to sequence exomes from individuals with diseases of unclear genetic etiology for the purpose of clinical diagnostics. However, even upon detection of candidate *de novo* variants, there is still insufficient information about the common and rare variants, which precludes a clear conclusion about the pathogenicity of the identified *de novo* variant and its role in disease (Acuna-Hidalgo et al., 2016). This limitation may be explained by the fact that *de novo* variants are usually heterozygous and may be either extremely rare or common. In cases of very rare *de novo* variants, the pathogenicity of the variant may be hard to prove as there are no more patients with the same phenotype and *de novo* variant. In cases of common *de novo* variants, the factors that determine the manifestations of variant pathogenicity may not be known, particularly if some individuals in the general population have the variant but do not have the genetic disease. However, irrespective of the rate of *de novo* variants, both types of variants may be scaled on the basis of relative fitness and natural selection.

The adaptedness depends on many factors; therefore, to assess whether a DNM is pathogenic or adaptive, and to understand why it occurs with a particular frequency in the population, it is necessary to examine the variant in appropriate conditions. Those include environment, parent age, genomic context, epigenetics, and other factors because all of them influence the value of mean relative fitness that increases monotonically, whereas the strength of selection decreases (Peck and Waxman, 2018).

The principal aim of this study was to elucidate the rate of occurring DNMs and to determine how these mutations are distributed in the exomes of general Lithuanian population. We also examined whether the frequency of these mutations was affected by the composition or structural parameters of the sequences in which they occurred and other factors that could influence the mechanisms underlying the formation of these DNMs. Finally we sought to establish whether DNMs emerged because of intensive pressure of natural selection on the functional regions. Although the distribution and intensity of DNMs have been subjects of many studies, they had not been explored previously in the Lithuanian population.

## Materials and Methods

In this study, we analyzed samples from Lithuanian population obtained from LITGEN project (LITGEN, 2011). The data set consisted of 49 trios with a total of 144 different individuals. Genomic DNA was extracted from venous blood using either phenol-chloroform extraction method or automated DNA extraction platform TECAN Freedom EVO^®^ (Tecan Schweiz AG, Switzerland) based on the paramagnetic particle method. Exomes were sequenced on a SOLiD 5500 sequencing system (75 bp reads). Sequencing data were processed and prepared by Lifescope software. Exomes were mapped according to the human reference genome build 19. An average read depth of sequencing was 38.5. BAM-formatted files of mother, father, and child generated by Lifescope were combined using SAMtools software for each trio.

*De novo* mutations were identified by two software programs: VarScan (Koboldt et al., 2012) and VarSeq^TM^. A potential variant was deemed to be a DNM if it was identified in the offspring but was not present in either of the parents at the same position. Overall, 1,752 and 4,756 DNMs were detected by VarScan and VarSeq^TM^, respectively. To discard false-positive *de novo* calls, when it was unknown whether all of the individuals in the trio were identified correctly, conservative filters on detected DNM quality parameters were applied as follows: (1) genotype quality of the individual ≥50; (2) number of reads at each site >20. SnpSift software was used to apply these filters on the data generated by VarScan. Data generated by VarSeq^TM^ software were filtered by choosing the same filtering parameters in the *Trio Workflow* segment. Furthermore, in order to discard the remaining variants that were somatic (only present in a fraction of the sequenced blood cells) with low allele balance or sequencing artifacts, DNMs were filtered by setting a threshold for the observed fraction of the reads in individuals with the alternative allele (the allele balance) for the trio [0.3; 0.7] (Kong et al., 2012; Besenbacher et al., 2015; Francioli et al., 2015). In addition, all possible identified and filtered *de novo* single nucleotide variants were manually reviewed by Integrative Genomics Viewer (Robinson et al., 2011). Due to the large number of identified DNMs, for the validation of variants by Sanger sequencing, 51 *de novo* single nucleotide variants were randomly selected. Sanger sequencing was performed using an ABI PRISM 3130xl Genetic Analyzer. All filtered and manually reviewed DNMs identified by VarScan (*N* = 95) and by VarSeq^TM^ (*N* = 84) were annotated using ANNOVAR (Butkiewicz and Bush, 2016; Wang et al., 2010). For the analysis of proteins interactions, STRING software (Szklarczyk et al., 2017) was used. As in the case of exome mapping, annotations were performed using hg19 reference human genome.

The probability that a calling position was a DNM in the trio was calculated independently for each trio. As described in a previous reference (Besenbacher et al., 2015), the *de novo* rate per position per generation (PPPG) was calculated as follows:

$$De\;novo\;{\text{rate}}_{\text{PPPG}}\;=\;\frac{\sum_{\text{i=1}}^{f}n_{\text{i}}}{2\sum_{\text{i=1}}^{f}\sum_{\text{j=1}}^{N}P_{\text{j}}^{i}(de\;novo)}$$

where *f* is the number of trios and *N* is the number of callable sites, that potentially can be identified as *de novo* sites for each trio separately, regardless of the sequencing depth. This number varies depending on trio. *n_i_* is the number of identified DNMs for trio *i*. The probability $P_{\text{j}}^{i}$ (*de novos* ingle nucleotide) for the called single nucleotide site *j* and family *i* to be mutated was calculated as follows:

(1) $$P_{\text{j}}^{i}(de\;novo\text{ single nucleotide})\;=\;P_{\text{j}}^{i}(C_{\text{Hetero}}|M_{\text{HomR}},F_{\text{HomR}})+P_{\text{j}}^{i}(C_{\text{Hetero}}|M_{\text{HomA}},F_{\text{HomA}})$$

The probability $P_{\text{j}}^{i}$ (*de novo* indel) for the called indel site *j* and family *i* to be mutated was calculated as:

$$P_{\text{j}}^{i}(de\;novo\text{ indel})\;=\;P_{\text{j}}^{i}(C_{\text{HomR}}|M_{\text{HomA}},F_{\text{HomA}})$$

where *C, M*, and *F* stand for offspring, mother, and father, respectively, and *Hetero, HomR*, and *HomA* denote heterozygous, homozygous for reference, and homozygous for alternative allele, respectively. The probability $P_{i}^{j}$ *(de novo)* was calculated with respect to the sequencing coverage. Confidence intervals for rate estimates were calculated as for binomial proportions. For the estimation of the DNM rate and for further calculations, we used R package (version 3.4.3) (R Core Team, 2013).

In order to test the hypothesis that variations in DNM rate across different regions of the genome could be explained by intrinsic characteristics of the genomic region itself and parent age, linear regression analysis was performed, for which the “secondary” annotation of each DNM was carried out using data from ENCODE (ENCODE Project Consortium, 2012) and LITGEN (LITGEN, 2011) projects. First, according to a previous study (Besenbacher et al., 2015), in order to collect records regarding the genomic landscape of the identified DNMs, lymphoblastoid cell lines (LCL and GM12878) (ENCODE Project Consortium, 2012) were chosen. Data were collected for:

(1) expression rates (eQTL) (ENCODE Project Consortium, 2012; Lappalainen et al., 2013; GTEx Consortium et al., 2017) in different tissues. According to expression of regions with DNMs were divided into positions with specific and non-specific expression;
(2) measurements of DNase1 hypersensitivity sites (DHS). DHS status was assigned 0 if outside DHS peak and 1 if within;
(3) measurements of context of CpG islands. If DNM was within CpG islands a status of position was assigned 1; if outside – 0;
(4) three histone marks (H3K27ac, H3K4me1, and H3K4me3) from the ENCODE project. If DNM was in position marked with histone it was assigned with 1 and if not – 0;
(5) GERPP++ conservation values were collected using ANNOVAR annotation tool. According to conservation values positions with DNMs were assigned into conservative (GERP++ score >12) and non-conservative positions (GERP++ score <12) (Davydov et al., 2010; ENCODE Project Consortium, 2012). Based on questionnaire records from LITGEN project, data on parental age were collected. After collection of parameters for each trio a number of positions with each parameter was calculated. Then a correlation analysis followed by linear regression modeling of DNM rate and parameters was performed.

## Results

**FIGURE 1:**
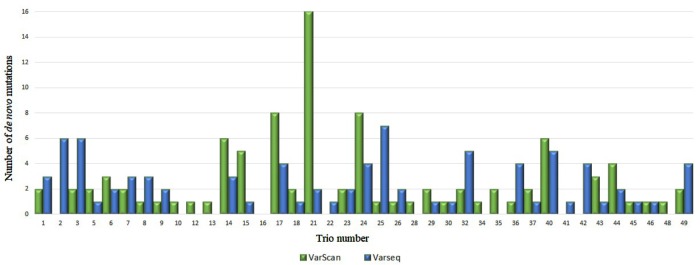
Comparison of *de novo* single nucleotide variants identified by VarScan (blue) and VarSeq^TM^ (green) software.

After DNM analysis, an exceptionally high number of DNMs were identified for two trios (nos. 4 and 21): 113 and 123 (by VarScan and VarSeq^TM^, respectively) and 16 (VarScan). These findings prompted us to test biological paternity, which was rejected for trio no. 4 and confirmed for trio no. 21. Thus, data for trio no. 4 were excluded from the study. In the final set of 48 trios, 95 DNMs were identified in 34 trios with VarScan software and 84 DNMs in 31 trios were identified with VarSeq^TM^ software (**Figure 1**). No DNMs were detected in 18 and 15 trios by VarScan and VarSeq^TM^, respectively. Of all DNMs identified by both software programs, only 5.37% of DNMs matched (three DNMs in *MEIS2, PGK1*, and *MT1B* genes). Each person had 1.9 (VarScan software) and 1.7 (VarSeq^TM^) DNMs on average.

**FIGURE 2:**
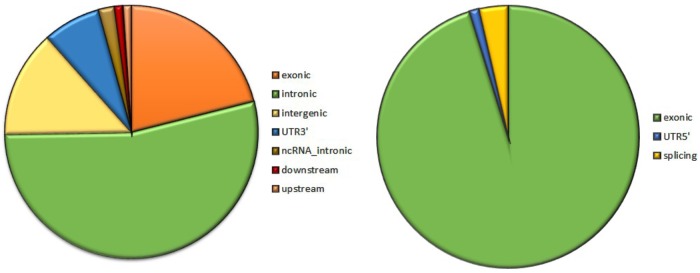
The composition of *de novo* mutations (DNMs) generated by VarScan (at left) and by VarSeq^TM^ (at right).

**FIGURE 3:**
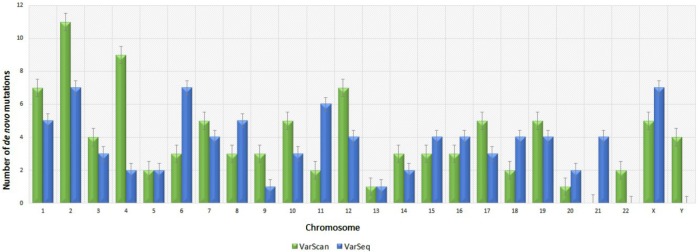
Distribution of number of *de novo* variants by chromosome according to the VarScan and VarSeq^TM^ generated data. Green bars represent DNMs identified by VarScan software, blue – by VarSeq^TM^. The error bars represent the standard error of the mean DNMs for each chromosome.

**FIGURE 4:**
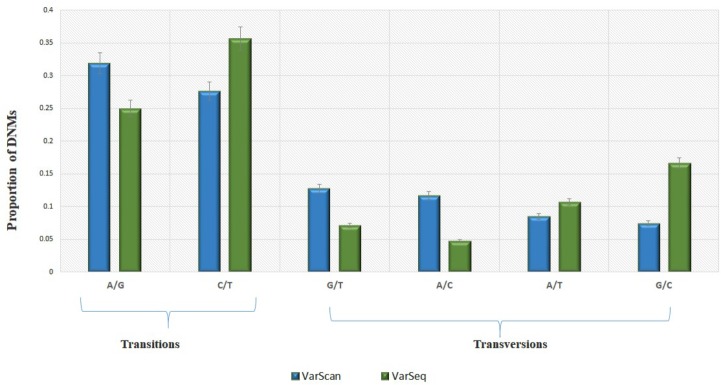
The molecular events underlying transitions occur more frequently than those leading to transversions, resulting in a ∼1.5-fold greater rate of transitions over transversions across the exome. Transition and transversion events identified by VarScan (green) and VarSeq^TM^ (blue) software. The error bars represent the standard error of the mean DNMs.

Analysis of 95 DNMs that were identified by VarScan software showed that 20 DNMs were exonic, including two stop-gain DNMs, seven synonymous DNMs, and 11 non-synonymous DNMs. Eighty novel mutations identified by VarSeq^TM^ were exonic, including 1 stop-gain DNM and 78 non-synonymous DNMs (**Figure 2**). The majority of DNMs identified by VarScan were in chromosomes 1, 2, 4, and 5, whereas VarSeq^TM^ identified DNMs predominantly in chromosomes 2, 6, 7, and 11. The number of identified DNMs did not correlate with the density of genes in the chromosomes (*R* = 0.09, *p*-value = 0.65 for VarScan and *R* = 6.73, *p*-value = 0.51 for VarSeq^TM^) or with the chromosome size (**Figure 3**). According to both software programs, the ratios of transitions and transversions were very similar: 1.44 and 1.47, respectively (**Figure 4**). However, differences in the structures of transitions were identified. Specifically, among DNMs identified by VarScan, there were more G/T and A/C changes, whereas among DNMs identified by VarSeq^TM^, there were more A/T and G/C changes.

The calculated rates of *de novo* single nucleotide mutations were 2.4 × 10^-8^ PPPG (95% confidence interval [CI]: 1.96 × 10^-8^–2.99 × 10^-8^) according to VarSeq^TM^ and 2.74 × 10^-8^per nucleotide per generation (95% CI: 2.24 × 10^-8^–3.35 × 10^-8^) according to VarScan.

Three *de novo* indels in three trios were identified by the VarScan algorithm in chromosomes 6 and 11. The calculated rate of *de novo* indels in the genome was 1.77 × 10^-8^ (95% CI: 6.03 × 10^-9^–5.2 × 10^-8^) PPPG. Notably, all *de novo* indels were “reversible,” i.e., parents had new variants in the genome, and their children had *de novo* variants based on the reference genome with the 37.5 mean value of depth of sequencing and 50 genotype quality, respectively. However, these three DNMs were not selected for the validation by Sanger sequencing method thus remains a probability of overestimation of *de novo* indels nevertheless. *De novo* indels were C/T and A/G in the context of single nucleotides.

Linear regression modeling revealed that DNAse 1 hypersensitivity sites, context of CpG islands, GERPP++ conservation values, and expression levels explained ∼68–93% of DNM rates (**Table 1**). Neither epigenetic markers nor paternal age significantly correlated with the DNM rate. The models were established from the data obtained from VarScan only because there was no correlation between data from VarSeq^TM^ and intrinsic characteristics of the genomic region itself.

**Table 1:** The linear regression of the DNAaseI hypersensitivity sites, context of CpG islands, GERPP++ conservation values and expression level effect of on the rate of DNMs.

Response variable	Independent variable	β ± SE	*p*-Value	*R*^2^
DNM rate	DNAaseI site	2.83 × 10^-6^± 1.96 × 10^-7^	2.31 × 10^-13^	0.89
DNM rate	Conservative site	5.28 × 10^-6^± 5.67 × 10^-7^	2.45 × 10^-9^	0.88
DNM rate	Non conservative site	5.28 × 10^-6^± 5.67 × 10^-7^	1.49 × 10^-6^	0.8
DNM rate	Site rich with CpG	2.76 × 10^-6^± 4.94 × 10^-7^	8.93 × 10^-6^	0.68
DNM rate	Site with non-specific expression	2.05 × 10^-6^± 1.144 × 10^-7^	8.34 × 10^-14^	0.94

*β, regression coefficient; SE, standard error. The models were constructed from data obtained from VarScan only.*

### Functional Prediction of DNMs

**FIGURE 5:**
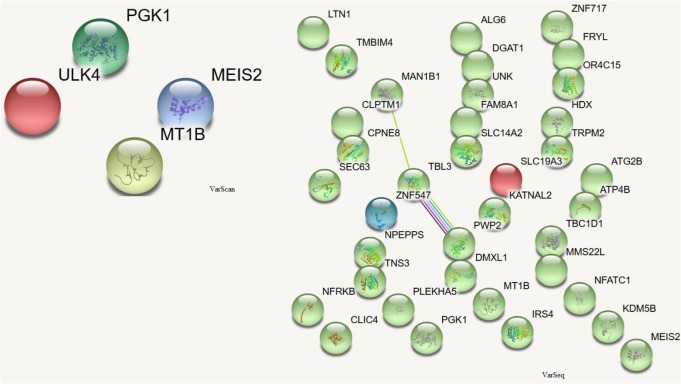
Protein-protein interactions (Szklarczyk et al., 2017) in genes harboring DNMs. DNMs identified by VarScan in genes coding proteins are at left, DNMs identified by VarSeq^TM^ – at right. Colored lines indicate a connection between proteins.

In order to assess which missense mutations were deleterious and altered the function of the affected protein by type, predicted categorical scores for the damage induced by DNMs were analyzed. The following 10 values were considered: polyphen HDIV and HVAR, LRT, PROVEAN, CADD, FATHMM, Mutation Taster, MutationAssessor, SIFT, Fathmm-MKL coding, and GERP++. Based on the predicted scores, four DNMs identified by VarScan as having six or more damaging or probably damaging predictions were selected. These stop-gain DNMs were in the *MEIS2* and *ULK4* genes, whereas non-synonymous DNMs were in the *MT1B* and *PGK1* genes. Proteins encoded by these genes are important for neuronal growth, endocytosis, and protection from the negative effects of heavy metals. These proteins participate in the release of the tumor blood vessel inhibitor angiostatin and in various signaling pathways. There were no connections between the proteins encoded by these genes (**Figure 5**).

*De novo* mutations identified by VarSeq^TM^ were analyzed in more detail if they were predicted to be damaging or probably damaging by at least half of the prediction tools. There were 35 point mutations (see ??) in genes encoding proteins that were important for chromatin remodeling, regulation of cytoskeleton, cell growth and viability, cytoplasmic signaling pathways, and the initiation of neuronal responses triggering the perception of smell.

Among the proteins encoded by the DNM-affected genes, only CLPTM1, ZNF547, and DMXL1 were connected in some way (**Figure 5**).

## Discussion

In this study, we conducted a comprehensive analysis of the distribution of DNMs across different regions of the exome in the Lithuanian population. In total, 95 DNMs in 34 trios and 84 DNMs in 31 trios were detected using SOLiD 5500 sequencing technology by VarScan and VarSeq^TM^ algorithms, respectively. First of all we would like to notice that we chose VarScan for DNMs calling because according to (Warden et al., 2014) this algorithm results a list of variants, with high concordance (>97%) to high-quality variants called by the GATK UnifiedGenotyper and HaplotypeCaller. VarSeq^TM^ software was chosen because it is widely used tool for variant analysis both in researches and clinical analysis. Despite both algorithms being designed to search for DNMs in the offspring exome that were not present in either parent, the agreement between the two software programs for DNM analysis was only 5.37%. The VarScan algorithm had higher sensitivity (5.42%) for DNM detection before filtration than VarSeq^TM^ algorithm (1.77%) thus, we suspected that no tool succeeded in calling mutations because of high sensitivity which was always accompanied by low specificity. Therefore we suggest that a considerable improvement in the results might be achieved by combining the output of different tools (Sandmann et al., 2017).

Based on the generated data, the estimated single nucleotide DNM rate was between 2.4 × 10^-8^ and 2.74 × 10^-8^ and that of *de novo* indels was 1.77 × 10^-8^ PPPG, depending on the algorithm used. Our calculated DNM rate was higher than that reported in previous studies (Kong et al., 2010, 2012; Neale et al., 2012; Szamecz et al., 2014; Besenbacher et al., 2015; Francioli et al., 2015), in which it varied between 1.2 × 10^-8^ and 1.5 × 10^-8^ PPPG. The higher DNM rate in our study was reasonable because our study was based on exome data. Additionally, exomes exhibit significantly higher (by 30%) mutation rates than whole genomes because the base pair composition of the whole genome is different from that of exomes. Notably, exomes have an average GC content of approximately 50%, whereas that of the whole genome is approximately 40% (Neale et al., 2012). Methylated CpGs represent highly mutable sequences in humans due to the spontaneous deamination of cytosine bases (Neale et al., 2012). According to comparative genomics studies, the increased mutation rates at CpG-rich regions are thought to have evolved around the time of mammalian radiation (Francioli et al., 2015). During the divergence of species, CpG-rich exonic regions underwent increased mutation rates compared to those at non-coding DNA and turned into non-coding regions. Therefore, then the effect of CpG content decrease over time, the average rate of mutation decrease until it reaches the level present in the surrounding non-coding DNA (Subramanian and Kumar, 2003). However, whereas sequences in neutrally evolving regions of the genome have had sufficient time to equilibrate with respect to dinucleotide contexts, purifying selection has maintained hypermutable CpGs in functional regions (Subramanian and Kumar, 2003; Schmidt et al., 2008; Francioli et al., 2015). Therefore, because we found a higher DNM rate than that reported by other studies, we speculated that it might be at least partially due to the local sequence context and/or possible natural selection pressure on the exome. Accordingly, a linear regression model was applied, and we found that DNAse 1 hypersensitivity, context of CpG islands, GERPP++ conservation values, and expression level explained ∼68–93% of the DNM rate. These findings indicated that DNMs in the exome formed independently from the conservation of DNA sequences. However, the DNM rate was higher in genes whose products were non-specific and in transcriptionally active promoter-like regions.

In contrast to the results of other studies (Wong et al., 2016; Sandmann et al., 2017), we found that paternal age did not correlate with the DNM rate. These findings could be explained by the fact that the data set consisted of trios with similar parent ages and that only a small portion (∼1.5%) of the whole genome was analyzed. Based on these parameters, each person had only 1.9 (VarScan) or 1.7 (VarSeq^TM^) DNMs on average compared to 40–82 in the whole genome (Crow, 2000; Branciamore et al., 2010; Kong et al., 2012; Neale et al., 2012; Besenbacher et al., 2015; Francioli et al., 2015; Wong et al., 2016), whereas the number of *de novo* indels in the coding sequence was similar to that identified in (Front Line Genomics, 2017).

The results of our extensive functional analysis of annotations revealed that out of all identified DNMs, 4 (VarScan) and 35 (VarSeq^TM^) variants were likely to be pathogenic DNMs. The difference in the number of pathogenic DNMs may be explained by the fact that depending on the algorithm used for the identification of DNMs, the share of DNMs in coding sequences differed significantly. For example, 21.05% of DNMs identified by VarScan software were exonic, whereas 95.24% of those identified by VarSeq^TM^ software were exonic. These pathogenic DNMs were in the genes encoding proteins that are essential for chromatin modeling, regulation of the cytoskeleton, modulation of cell growth and vitality, function of cytoplasmic signaling pathways, and initiation of neuronal response. Despite those DNMs being considered pathogenic, all individuals taking the survey identified themselves as genetically “healthy.” Therefore, this result indicated that despite the putative pathogenicity of DNMs, the genomes in which DNMs were located obviously tolerated such changes, so that disease manifestations were often not pronounced. According to Szamecz et al. (2014), the more often DNMs occur in conserved genetic positions, the stronger are the effects of natural selection on genetic changes through compensatory mechanisms of genome protection. The harmful effects of the variants can be mitigated in four ways. Some genes can tolerate truncated variants of proteins because their functional effects are masked by incomplete expression, compensatory variants, or low functional significance of the truncation (Bartha et al., 2015). In contrast, gene changes associated with non-synonymous DNMs are compensated through the mechanism of useful mutation accumulation throughout the whole genome (Szamecz et al., 2014). It suggests that in these cases, the pathogenic mutations are not deleterious enough to reduce mean fitness and therefore, they persist for longer in many generations being shaped by natural selection.

In summary, our analysis of the distribution of DNMs and of their genetic and epigenetic context provided insights into genetic variation of Lithuanian genome. Based on these findings, additional studies in patient groups with genetic diseases may facilitate our ability to distinguish certain pathogenic DNMs from the tolerated background DNMs and to identify reliable causative DNMs. However, the principal limitation of this study was in that we did not examine variation in non-coding and regulatory gene regions. This information could contribute to the elucidation of possible mechanisms of DNM formation that still remain insufficiently clear.

## Accession Codes

Sequence data have been deposited at the European Nucleotide Archive (ENA), under accession PRJEB25864 (ERP107829).

## Ethics Statement

This study was carried out in accordance with the recommendations of permission, Vilnius Regional Ethics Committee for Biomedical Research. The protocol was approved by the Vilnius Regional Ethics Committee for Biomedical Research. All subjects gave written informed consent in accordance with the Declaration of Helsinki.

## Author Contributions

LP performed data analysis and prepared the manuscript. AJ calculated the rate of *de novo* mutations. Sequencing of trios exomes was performed by LA and IK. VK was the principal investigator.

## Conflict of Interest Statement

The authors declare that the research was conducted in the absence of any commercial or financial relationships that could be construed as a potential conflict of interest.
