# Supplementary material for: Insights Into de novo Mutation Variation in Lithuanian Exome
Source: Front Genet. 2018 Aug 14;9:315. doi: 10.3389/fgene.2018.00315 (PMC6102505; doi:10.3389/fgene.2018.00315)
Supplement: Supplementary file 1 [file Table_1.DOCX]

Supplementary Table 1. The annotations of genes which *de novo* variants were generated by VarSeq™ and annotated as a “pathogenic”. NA – data not available.

| Gene | Change in DNA | Change in protein | Number of identification | Gene’s annotation |
| --- | --- | --- | --- | --- |
| *SLC19A3* | c.C1332G | p.S444R | rs863224204, CM133449 | Eencodes a ubiquitously expressed transmembrane thiamine transporter that lacks folate transport activity. |
| *KATNAL2* | c.C652G | p.L218V | NA | Gene include microtubule binding and microtubule-severing ATPase activity |
| *TBL3* | c.T1667C | p.L556P | NA | Its related pathways are rRNA processing in the nucleus and cytosol and Gene Expression. GO annotations related to this gene include poly(A) RNA binding and snoRNA binding. |
| *TMBIM4* | c.G464+1A |  | NA | Diseases associated with TMBIM4 include Venezuelan Hemorrhagic Fever and Lassa Fever. |
| *ATG2B* | c.A547G | p.K183E | NA | Gene encodes a protein required for autophagy. The encoded protein is involved in autophagosome formation. A germline duplication of a region that includes this gene is associated with predisposition to myeloid malignancies. |
| *ZNF547* | c.G1078C | p.A360P | NA | May be involved in transcriptional regulation. |
| *UNK* | c.C232T | p.R78W | rs764970108 | Acts as a translation repressor and controls a translationally regulated cell morphology program to ensure proper structuring of the nervous system. Translational control depends on recognition of its binding element within target mRNAs which consists of a mandatory UAG trimer upstream of a U/A-rich motif. |
| *ALG6* | c.A170AG | p.Y57C | NA | Gene encodes a member of the ALG6/ALG8 glucosyltransferase family. The encoded protein catalyzes the addition of the first glucose residue to the growing lipid-linked oligosaccharide precursor of N-linked glycosylation. |
| *ZNF717* | c.T65C | p.V22A | rs75138373 | This gene encodes a Kruppel-associated box (KRAB) zinc-finger protein, which belongs to a large group of transcriptional regulators in mammals. These proteins bind nucleic acids and play important roles in various cellular functions, including cell proliferation, differentiation and apoptosis, and in regulating viral replication and transcription. |
| *FAM8A1* | c.T863C | p.I288T | rs111827800 | Family With Sequence Similarity 8 Member A1, Autosomal Highly Conserved Protein |
| *ATP4B* | c.C203T | p.P68L | rs531698043 | The protein encoded by this gene belongs to a family of P-type cation-transporting ATPases. |
| *HDX* | c.T1739C | p.V580A | NA | Highly Divergent Homeobox |
| *PGK1* | c.A77G | p.N26S | rs201924559 | The protein encoded by this gene is a glycolytic enzyme that catalyzes the conversion of 1,3-diphosphoglycerate to 3-phosphoglycerate. The encoded protein may also act as a cofactor for polymerase alpha. Additionally, this protein is secreted by tumor cells where it participates in angiogenesis by functioning to reduce disulfide bonds in the serine protease, plasmin, which consequently leads to the release of the tumor blood vessel inhibitor angiostatin. The encoded protein has been identified as a moonlighting protein based on its ability to perform mechanistically distinct functions. Deficiency of the enzyme is associated with a wide range of clinical phenotypes hemolytic anemia and neurological impairment. |
| *KDM5B* | c.G3475C | p.E1159Q | rs373778742 | Lysine-specific capable of demethylating tri-, di- and monomethylated lysine 4 of histone H3histone demethylase. |
| *MMS22L* | c.T199A | p.F67I | rs758142671 | The protein encoded by this gene forms a complex with tonsoku-like, DNA repair protein (TONSL), and this complex recognizes and repairs DNA double-strand breaks at sites of stalled or collapsed replication forks. The encoded protein also can bind with the histone-associated protein NFKBIL2 to help regulate the chromatin state at stalled replication forks. |
| *MAN1B1* | c.G757A | p.V253M | NA | Gene encodes an enzyme belonging to the glycosyl hydrolase 47 family. This enzyme functions in N-glycan biosynthesis, and is a class I alpha-1,2-mannosidase that specifically converts Man9GlcNAc to Man8GlcNAc isomer B. |
| *NFATC1* | c.C962A | p.T321N | NA | The product of this gene is a component of the nuclear factor of activated T cells DNA-binding transcription complex. The product of this gene is an inducible nuclear component. It functions as a major molecular target for the immunosuppressive drugs such as cyclosporin A. |
| *DGAT1* | c.A797G | p.N266S | rs146196839 | Gene encodes a multipass transmembrane protein that functions as a key metabolic enzyme. Activity of this protein may be associated with obesity and other metabolic diseases. |
| *IRS4* | c.C2242T | p.P748S | NA | Gene encodes the insulin receptor substrate 4, a cytoplasmic protein that contains many potential tyrosine and serine/threonine phosphorylation sites. The IRS4 protein is phosphorylated by the insulin receptor tyrosine kinase upon receptor stimulation. Acts as an interface between multiple growth factor receptors possessing tyrosine kinase activity, such as insulin receptor, IGF1R and FGFR1, and a complex network of intracellular signaling molecules containing SH2 domains. |
| *CLIC4* | c.G403C | p.E135Q | NA | A member of the p64 family; the gene is expressed in many tissues and exhibits a intracellular vesicular pattern in Panc-1 cells (pancreatic cancer cells), Can insert into membranes and form poorly selective ion channels that may also transport chloride ions. |
| *SEC63* | c.G319C | p.E107Q | NA | The Sec61 complex is the central component of the protein translocation apparatus of the endoplasmic reticulum (ER) membrane. The protein encoded by this gene and SEC62 protein are found to be associated with ribosome-free SEC61 complex. The encoded protein is an integral membrane protein located in the rough ER. Required for integral membrane and secreted preprotein translocation across the endoplasmic reticulum membrane |
| *CLPTM1* | c.T614C | p.F205S | NA | Transmembrane Protein. Diseases associated with CLPTM1 include Cleft Lip. An important paralog of this gene is CLPTM1L.May play a role in T-cell development. |
| *MEIS2* | c.C1006T | p.Q336X | NA | Gene encodes a homeobox protein belonging to the TALE ('three amino acid loop extension') family of homeodomain-containing proteins. TALE homeobox proteins are highly conserved transcription regulators, and several members have been shown to be essential contributors to developmental programs. |
| *TBC1D1* | c.G887A | p.G296D | rs145932557 | May act as a GTPase-activating protein for Rab family protein(s). May play a role in the cell cycle and differentiation of various tissues. Involved in the trafficking and translocation of GLUT4-containing vesicles and insulin-stimulated glucose uptake into cells. |
| *NFRKB* | c.T2507A | p.I836N | NA | Nuclear Factor Related To KappaB Binding Protein, Binds to the DNA consensus sequence 5-GGGGAATCTCC-3. Putative regulatory component of the chromatin remodeling INO80 complex which is involved in transcriptional regulation, DNA replication and probably DNA repair. |
| *SLC14A2* | c.G1183A | p.V395M | rs781119545 | The protein encoded by this gene belongs to the urea transporter family. In mammalian cells, urea is the chief end product of nitrogen catabolism, and plays an important role in the urinary concentration mechanism. |
| *LTN1* | c.A378T | p.K126N | NA | LTN1 functions as an E3 ubiquitin ligase. Among its related pathways are Innate Immune System and Class I MHC mediated antigen processing and presentation. GO annotations related to this gene include binding and ubiquitin-protein transferase activity. |
| *PLEKHA5* | c.G350A | p.R117Q | rs144183813 | Its related pathways are PI Metabolism and Metabolism. GO annotations related to this gene include phosphatidylinositol-3-phosphate binding and phosphatidylinositol-3,5-bisphosphate binding. |
| *FAM8A1* | c.T863C | p.I288T | rs111827800 | Family With Sequence Similarity 8 Member A1 |
| *NPEPPS* | c.T1298G | p.F433C | rs200616431 | Gene encodes the puromycin-sensitive aminopeptidase, a zinc metallopeptidase which hydrolyzes amino acids from the N-terminus of its substrate. Its related pathways are Innate Immune System and Class I MHC mediated antigen processing and presentation. Involved in proteolytic events essential for cell growth and viability. May act as regulator of neuropeptide activity. Plays a role in the antigen-processing pathway for MHC class I molecules. |
| *TNS3* | c.C3292G | p.L1098V | rs188962919 | Its related pathways are MET promotes cell motility and Signaling by GPCR. GO annotations related to this gene include phosphatase activity. May play a role in actin remodeling. Involved in the dissociation of the integrin-tensin-actin complex. EGF activates TNS4 and down-regulates TNS3 which results in capping the tail of ITGB1. Seems to be involved in mammary cell migration. May be involved in cell migration and bone development. |
| *MT1B* | c.G138T | p.Q46H | NA |  |
| *TRPM2* | c.A2251 | p.N751D | rs771593421 | The protein encoded by this gene forms a tetrameric cation channel that is permeable to calcium, sodium, and potassium and is regulated by free intracellular ADP-ribose. The encoded protein is activated by oxidative stress and confers susceptibility to cell death. |
| *OR4C15* | c.C299T | p.T100I | rs753330230 | Olfactory receptors interact with odorant molecules in the nose, to initiate a neuronal response that triggers the perception of a smell. |
| *DMXL1* | c.A6776 | p.Q2259L | NA | Gene is expressed in many tissue types including several types of eye tissue, and it has been associated with ocular phenotypes. In addition, it is upregulated in cultured cells that overexpress growth factor independence 1B, a transcription factor that is essential for hematopoietic cell development. |
| *PWP2* | c.C146T: | p.T49M | rs371578994 | Diseases associated with PWP2 include Holoprosencephaly 1. Among its related pathways are rRNA processing in the nucleus and cytosol and Gene Expression. GO annotations related to this gene include poly(A) RNA binding and snoRNA binding. |
| *FRYL* | c.A1483G | p.T495A | NA | O annotations related to this gene include binding plays a key role in maintaining the integrity of polarized cell extensions during morphogenesis, regulates the actin cytoskeleton and plays a key role in patterning sensory neuron dendritic fields by promoting avoidance between homologous dendrites as well as by limiting dendritic branching (By similarity). May function as a transcriptional activator. |
| *CPNE8* | c.G410A | p.G137E | NA | Copine 8, Calcium-dependent membrane-binding proteins may regulate molecular events at the interface of the cell membrane and cytoplasm. This gene is one of several genes that encode a calcium-dependent protein containing two N-terminal type II C2 domains and an integrin A domain-like sequence in the C-terminus. Probable calcium-dependent phospholipid-binding protein that may play a role in calcium-mediated intracellular processes. |
